# N-3 Polyunsaturated Fatty Acids Prevent Diabetic Retinopathy by Inhibition of Retinal Vascular Damage and Enhanced Endothelial Progenitor Cell Reparative Function

**DOI:** 10.1371/journal.pone.0055177

**Published:** 2013-01-29

**Authors:** Maria Tikhonenko, Todd A. Lydic, Madalina Opreanu, Sergio Li Calzi, Svetlana Bozack, Kelly M. McSorley, Andrew L. Sochacki, Matthew S. Faber, Sugata Hazra, Shane Duclos, Dennis Guberski, Gavin E. Reid, Maria B. Grant, Julia V. Busik

**Affiliations:** 1 Department of Physiology, Michigan State University, East Lansing, Michigan, United States of America; 2 Department of Microbiology and Molecular Genetics, Michigan State University, East Lansing, Michigan, United States of America; 3 Department of Chemistry and Biochemistry and Molecular Biology, Michigan State University, East Lansing, Michigan, United States of America; 4 Biomedical Research Models, Inc., Worcester, Massachusetts, United States of America; 5 Department of Pharmacology and Therapeutics University of Florida, Gainesville, Florida, United States of America; University of Padova, Medical School, Italy

## Abstract

**Objective:**

The vasodegenerative phase of diabetic retinopathy is characterized by not only retinal vascular degeneration but also inadequate vascular repair due to compromised bone marrow derived endothelial progenitor cells (EPCs). We propose that n-3 polyunsaturated fatty acid (PUFA) deficiency in diabetes results in activation of the central enzyme of sphingolipid metabolism, acid sphingomyelinase (ASM) and that ASM represents a molecular metabolic link connecting the initial damage in the retina and the dysfunction of EPCs.

**Research Design and Methods:**

Type 2 diabetic rats on control or docosahexaenoic acid (DHA)-rich diet were studied. The number of acellular capillaries in the retinas was assessed by trypsin digest. mRNA levels of interleukin (IL)-1β, IL-6, intracellular adhesion molecule (ICAM)-1 in the retinas from diabetic animals were compared to controls and ASM protein was assessed by western analysis. EPCs were isolated from blood and bone marrow and their numbers and ability to form colonies *in vitro*, ASM activity and lipid profiles were determined.

**Results:**

DHA-rich diet prevented diabetes-induced increase in the number of retinal acellular capillaries and significantly enhanced the life span of type 2 diabetic animals. DHA-rich diet blocked upregulation of ASM and other inflammatory markers in diabetic retina and prevented the increase in ASM activity in EPCs, normalized the numbers of circulating EPCs and improved EPC colony formation.

**Conclusions:**

In a type 2 diabetes animal model, DHA-rich diet fully prevented retinal vascular pathology through inhibition of ASM in both retina and EPCs, leading to a concomitant suppression of retinal inflammation and correction of EPC number and function.

## Introduction

In diabetes, dysfunction of the endothelium is a key factor in development of vascular complications [1], although nonvascular retinal tissue also plays an important role in the development of retinal pathology [2]. During the vasodegenerative stage of diabetic retinopathy (DR), capillary components such as pericytes and endothelial cells die prematurely [3]. This diabetes-induced injury of the retinal microvessels leads to the hallmark features of DR: increased vascular permeability, acellular capillary formation and non-perfusion, and ultimately neovascularization in the proliferative stage of DR [3].

To counteract diabetes-induced endothelial cell injury, several endogenous repair mechanisms are available. Traditionally, microvascular repair has been considered to be proliferation of the resident vasculature [4]. However, there is a significant body of evidence that advocates that cells identified as EPCs are a subpopulation of progenitor cells that play a critical role in endothelial repair and maintenance [5]. EPCs generated in the bone marrow are mobilized into the blood and recruited to areas of injury where they can incorporate into injured vessels and differentiate into endothelial cells to replace compromised endothelium but also provide paracrine support to the resident vasculature [6]. Accumulating evidence suggests that diabetic EPCs lose the ability to perform these functions, contributing to the development of the degenerative stage of DR [7]. Our recent report revealed that diabetic bone marrow neuropathy affects EPC release and function and precedes retinal vascular degeneration in diabetes [8] providing additional evidence for the critical role of these cells in disease pathogenesis.

We and others have shown that activated ASM participates in inflammatory cytokine signaling [9], [10], particularly in endothelial cells [11]. ASM is a critical enzyme that catalyzes hydrolysis of sphingomyelin (SM) to ceramide (Cer), and is rapidly activated in conditions of inflammation or stress [12].

N-3 PUFAs and especially DHA have a unique ability to modulate capillary integrity, neovascularization and inflammation in the retina [13]. DHA is decreased in diabetic retina [14], [15] and plasma [16]. We and others demonstrated that increased dietary intake of n-3 PUFAs prevents retinopathy in both type 1 diabetes [17] and a model of retinopathy of prematurity [18]. There are, however, no studies addressing the role of n-3 PUFAs in type 2 diabetes. Recently, we demonstrated that DHA acts through inhibition of the ASM pathway to prevent retinal endothelial cell activation by inflammatory cytokines {Opreanu, 2010 #896}. EPCs are endothelial lineage cells and may be affected by the same mechanism that influences retinal endothelial cells. Despite the recognized EPC dysfunction in diabetes that prevents vascular repair and regeneration, there is a dearth of information on the effects of fatty acids on EPCs and particularly on the effects of n-3 PUFA of EPC function. In this study of type 2 diabetes, we propose that n-3 PUFA-mediated ASM downregulation improves diabetic retinopathy outcomes through inhibition of retinal vascular damage and enhanced EPC function leading to retinal vascular repair.

## Results

### Effect of DHA rich diet on formation of acellular capillaries in type 2 diabetic rats

**Figure 1:**
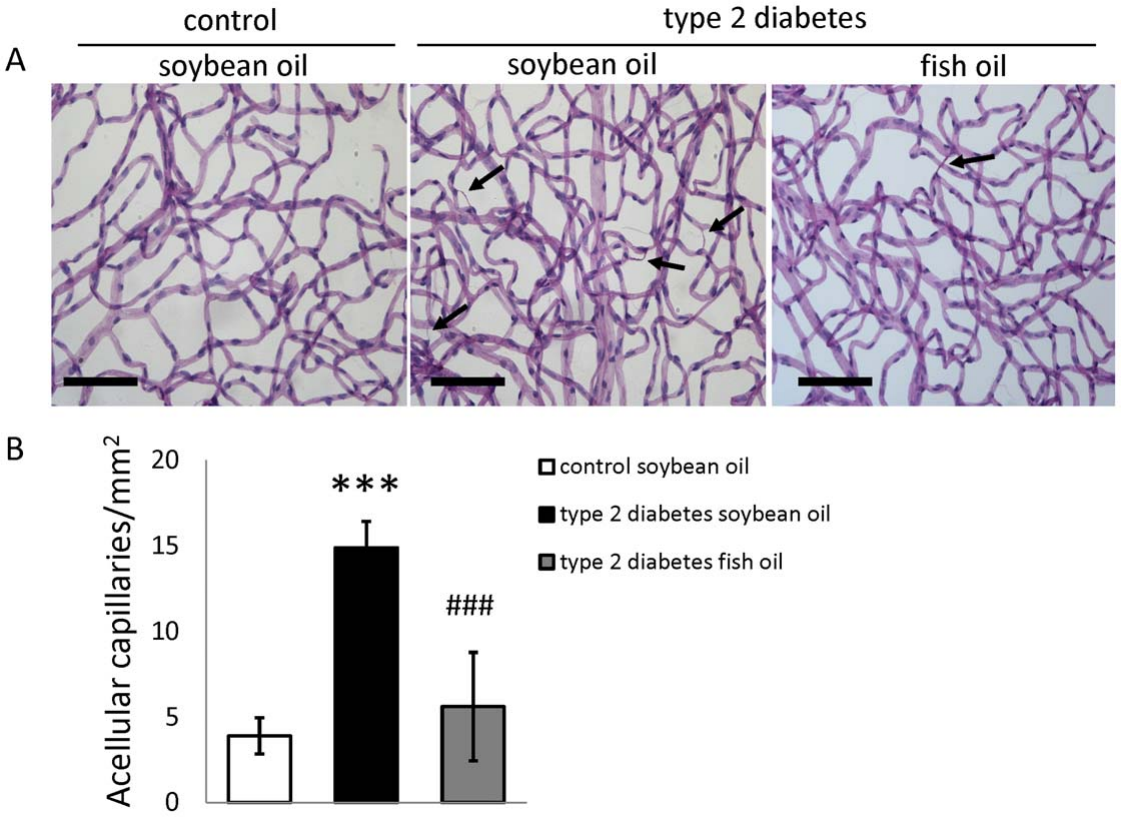
DHA-enriched fish oil diet prevents the formation of acellular capillaries in type 2 diabetes model. A) Trypsin digests of rat retinas stained with hematoxylin and periodic acid–Schiff. Representative regions around the mid-retina used for counting are shown. Bars,10 µM. Significantly increased number of acellular capillaries (black arrows) was observed in retinal vasculature isolated from diabetic animals compared with control. No significant difference was found between diabetic animals fed DHA diet and control animals. (B) Quantification of acellular capillaries in n = 9 in control and diabetes soybean diet group, n = 4 in diabetes DHA diet group. *** = p<0.001 compared to control animals group and ^###^ = p<0.001 compared to diabetic animals fed with soybean oil diet.

To determine whether a DHA enriched diet has a protective effect against the development of retinal vascular pathology in type 2 diabetes, Bio-Breeding Zucker diabetic rats (BBZDR/Wor) and their age-matched non-diabetic BBDR littermates were used. Retinal vasculature was isolated from diabetic rats subjected to either DHA enriched diet or standard rodent diet with soybean as a source of fat for 5–7 months. In agreement with previous reports, the number of acellular capillaries was dramatically increased in retinas from type 2 diabetic rats fed the control diet. However, the number of acellular capillaries in the retinas from diabetic rats fed a DHA enriched diet was not significantly different from control retinas (Fig. 1).

### Effect of DHA rich diet on retinal inflammatory markers in control and diabetic rats

**Figure 2:**
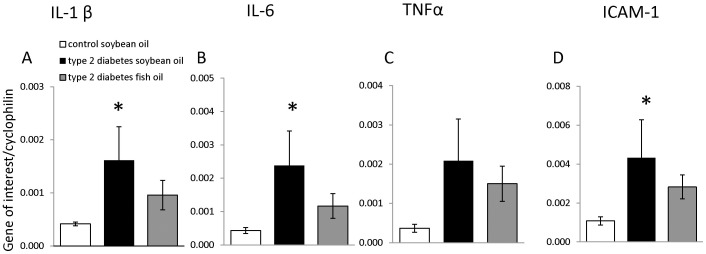
DHA-enriched fish oil diet prevents increase in IL-1β, IL-6, TNFα and ICAM gene expression in type 2 diabetes model. Gene expression of pro-inflammatory and pro-angiogenic mediators was assessed by real-time qPCR using the whole retina. A significant increase in IL-1β, IL-6, TNFα and ICAM levels was observed in diabetic rats fed a standard diet compared to control rats. DHA-enriched diet prevented these levels from reaching significant increase. n = 10 controls, n = 4 diabetes soybean oil, n = 8 diabetes fish oil. Data are expressed as the mean ± SE. * = p<0.05 compared to control animals group.

IL-1β, IL-6, ICAM-1 mRNA levels were increased in the retinas from type 2 diabetic animals. Importantly, the DHA-rich diet prevented the increases in IL-1β, IL-6, and ICAM-1expression (Fig. 2). Tumor necrosis factor α (TNFα) levels showed a trend towards an increase in diabetic animals fed control diet, but this was not statistically significant. We performed the measurements of the chemo attractants, Monocyte chemoattractant protein-1(MCP-1) and Stromal cell-derived factor-1 (SDF-1), and growth factors, Vascular endothelial growth factor (VEGF) and Pigment epithelium-derived factor (PEDF), but found high variability in the expression level of these factors with no differences between control, diabetes and treatment groups (data not shown).

**Figure 3:**
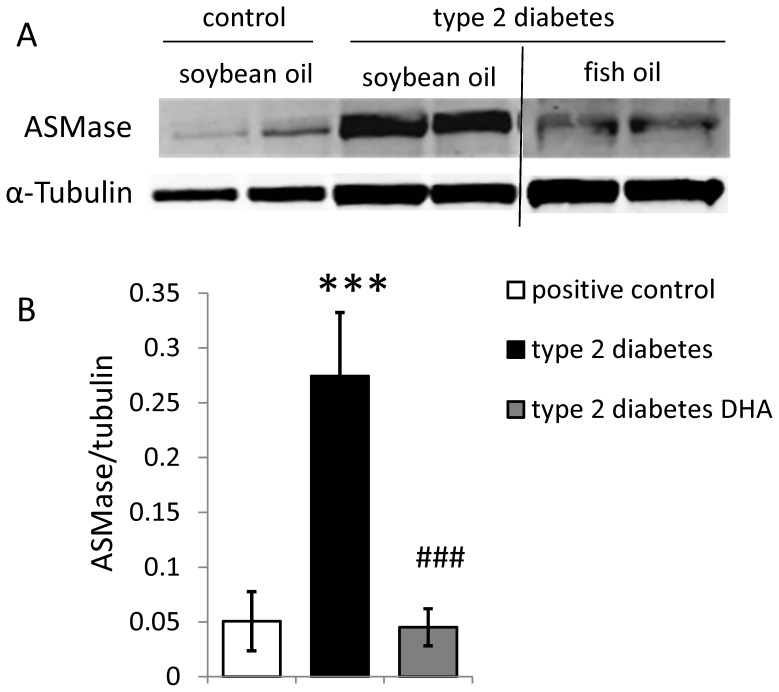
DHA-enriched fish oil diet normalizes the ASM protein levels in the whole retinas of type 2 diabetic rats. (A) Retinal acid sphingomyelinase protein levels were assessed by Western blot analysis. (B) Quantification of 4 rats/condition is shown in and normalized by tubulin (loading control). *** = p<0.001 compared to control animals group and ^###^ = p<0.001 compared to diabetic animals fed with soybean oil diet.

We have previously identified ASM as an important modulator of the inflammatory response in the retinal vasculature [11]. In this study we observed a dramatic increase in ASM protein levels in the retinas from diabetic animals on control diet which was completely prevented in the retinas from the animals on the DHA rich diet (Fig. 3).

### Effect of DHA rich diet on EPC number and colony formation in control and diabetic animals

**Figure 4:**
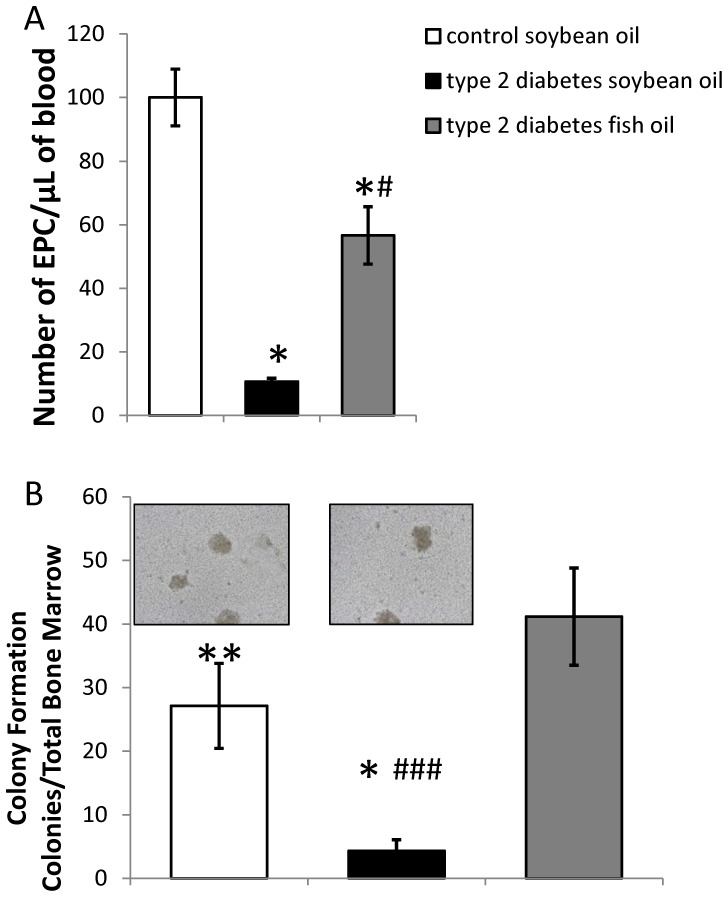
DHA-enriched fish oil diet normalizes circulating and bone marrow EPC numbers in type 2 diabetes model. A) EPCs were extracted by magnetic beads method from total peripheral blood. After 2 months of diabetes and feeding of the experimental diet, control and diabetic animals fed soybean or fish oil diet were used and the number of circulating EPCs. n = 8 controls, n = 5 in diabetes soybean oil diet, n = 7 in diabetes fish oil diet. * = p<0.05 compared to control animals group, ^#^ = p<0.05 compared to diabetic animals fed with soybean oil diet. B) EPCs were extracted by magnetic beads method from bone marrow and analyzed by colony formation assay. EPC colony formation was significantly decreased in diabetic rats compared to controls but corrected by DHA-enriched fish oil diet. The images of the colonies from control and diabetic animals are shown above the respective graphs. n = 4 in each group. * = p<0.05, ** = p<0.01 compared to control animals group, ^###^ = p<0.001 compared to diabetic animals fed with soybean oil diet.

To assess the effect of the DHA-rich diet on EPC reparative function, we first measured the number of EPCs in circulation in control animals, and diabetic animals fed the control or DHA supplemented diets. After 2 months of diabetes, the rats fed the control diet had significantly reduced number of EPCs in the peripheral blood (Fig. 4A). The number of EPCs in diabetic animals fed a DHA-rich diet was significantly higher compared to diabetic animals fed the control diet (Fig. 4A).

To test the function of EPCs, colony formation was evaluated. EPCs were extracted from the bone marrow of control rats and type 2 diabetic rats following 2 months of diabetes on either the control diet or the DHA-rich diet. EPCs from diabetic animals fed the control diet demonstrated decreased colony formation *in vitro* which was prevented in diabetic animals on DHA-rich diet (Fig. 4B).

### ASM specific activity in bone marrow derived EPCs

**Figure 5:**
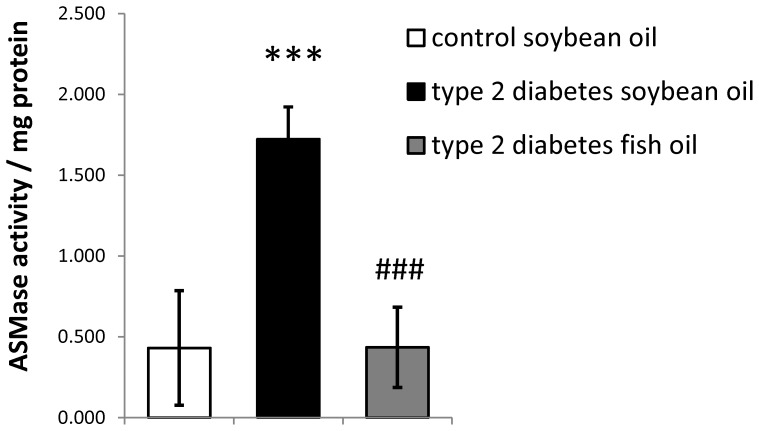
The increase in ASM activity observed in diabetic EPCs is prevented by DHA-rich fish oil diet. Diabetic bone marrow EPC showed a significant increase in ASM activity. However, the EPCs derived from diabetic animals fed DHA-rich fish oil diet showed reduced ASM activity that was similar to that observed in control rats. n = 8 controls, n = 3 in diabetes soybean oil diet, n = 5 in diabetes fish oil diet. *** = p<0.001 compared to control animals group and ^###^ = p<0.001 compared to diabetic animals fed with soybean oil diet.

Previously, we demonstrated that the protective effect of DHA in endothelial cells is due largely to inhibition of ASM activity. As EPCs are endothelial lineage cells, we next determined ASM activity in EPCs isolated from bone marrow of control and type 2 diabetic rats. EPCs from diabetic rats demonstrated increased ASM activity as compared to non-diabetic rats. Importantly, dietary supplementation with DHA reversed this elevation to control levels (Fig. 5).

### Sphingomyelin and ceramide levels in CD34^+^ cells from diabetic and control patients

Using tandem mass spectrometry analysis of SM and Cer species extracted from CD34^+^ EPCs, we found that d18:1/14:0 Cer; d18:1/16:0 and d18:1/24:1 Hexosyl Cer; d18:1/24:1 LactosylCer; d18:1/14:0 and d18:1/15:0 SM were significantly increased in diabetic EPCs compared to control. On the contrary, the levels of d18:1/18:0 Cer, d18:1/20:1 Hexosyl Cer; d18:1/22:0 and d18:1/24:0 LactosylCer; and d18:1/23:0 d18:1/24:0 SM were decreased in diabetic EPCs compared to control (Table 1).

**Table 1:** Sphingolipids in control and diabetic CD34+ EPCs.

Lipid species % of total	Control	Diabetes	Difference	P
Cer(d18:1/14:0)	0.13	0.52	**↑**	0.034
Cer(d18:1/16:0)	14.38	18.83		0.212
Cer(d18:1/18:0)	2.34	1.44	**↓**	0.043
Cer(d18:1/20:0)	2.36	2.26		0.855
Cer(d18:1/22:0)	14.33	11.37		0.276
Cer(d18:1/22:1)	0.70	0.48		0.567
Cer(d18:1/24:0)	22.82	19.90		0.551
Cer(d18:1/24:1)	17.39	18.52		0.535
Cer(d18:1/24:2)	5.14	4.83		0.670
Cer(d18:1/26:0)	7.17	10.50		0.692
Cer(d18:1/26:1)	1.45	1.96		0.495
Cer(d18:1/26:2)	0.88	0.66		0.721
Cer(d18:1/32:0)	0.13	0.09		0.764
Cer(d18:1/32:2)	0.57	0.07		0.316
Hex-Cer(d18:1/14:0)	0.58	0.28		0.650
Hex-Cer(d18:1/15:0)	2.25	3.54		0.561
Hex-Cer(d18:1/16:0)	21.23	34.34	**↑**	0.013
Hex-Cer(d18:1/18:0)	4.50	4.15		0.836
Hex-Cer(d18:1/19:0)	5.88	3.81		0.369
Hex-Cer(d18:1/20:0)	0.71	3.00		0.102
Hex-Cer(d18:1/20:1)	5.25	0.00	**↓**	0.002
Hex-Cer(d18:1/20:2)	4.69	0.16		0.318
Hex-Cer(d18:1/20:3)	0.17	0.86		0.452
Hex-Cer(d18:1/20:4)	7.07	0.74		0.184
Hex-Cer(d18:1/22:0)	6.52	4.31		0.170
Hex-Cer(d18:1/22:1)	0.22	0.39		0.624
Hex-Cer(d18:1/24:0)	16.51	14.09		0.591
Hex-Cer(d18:1/24:1)	6.45	23.30	**↑**	0.003
Hex-Cer(d18:1/24:1-OH)	1.10	0.53		0.640
Hex-Cer(d18:1/26:0)	0.16	0.31		0.669
Hex-Cer(d18:1/26:1)	7.13	1.85		0.093
Hex-Cer(d18:1/30:1)	0.25	0.67		0.565
LacCer(d18:1/14:0)	1.31	0.44		0.533
LacCer(d18:1/16:0)	68.74	63.70		0.443
LacCer(d18:1/18:0)	1.63	2.24		0.629
LacCer(d18:1/18:1)	1.27	1.78		0.656
LacCer(d18:1/20:0)	0.08	0.05		0.820
LacCer(d18:1/22:0)	11.53	5.66	**↓**	0.011
LacCer(d18:1/24:0)	9.70	6.04	**↓**	0.003
LacCer(d18:1/24:1)	4.99	18.89	**↑**	0.002
LacCer(d18:1/24:2)	0.49	0.39		0.833
SM(d18:1/14:0)	1.66	2.33	**↑**	0.009
SM(d18:1/15:0)	0.95	1.21	**↑**	0.004
SM(d18:1/16:0)	28.3	29.9		0.518
SM(d18:1/16:1)	1.72	1.68		0.668
SM(d18:1/18:0)	2.32	2.04		0.124
SM(d18:1/18:1)	0.76	0.68		0.450
SM(d18:1/20:0)	3.67	3.69		0.966
SM(d18:1/22:0)	17.7	15.1		0.166
SM(d18:1/23:0)	2.17	1.72	**↓**	0.021
SM(d18:1/24:0)	12.6	10.6	**↓**	0.023
SM(d18:1/24:1)	17.2	19.8		0.084
SM(d18:1/24:2)	4.61	4.51		0.794
SM(d18:1/26:0)	0.21	0.14		0.460
SM(d18:1/26:1)	0.47	0.44		0.759

Arrows pointing up indicate significant increase, arrows pointing down – significant decrease in diabetic EPCs as compared to control.

### Sphingomyelinase substrate specificity

**Figure 6:**
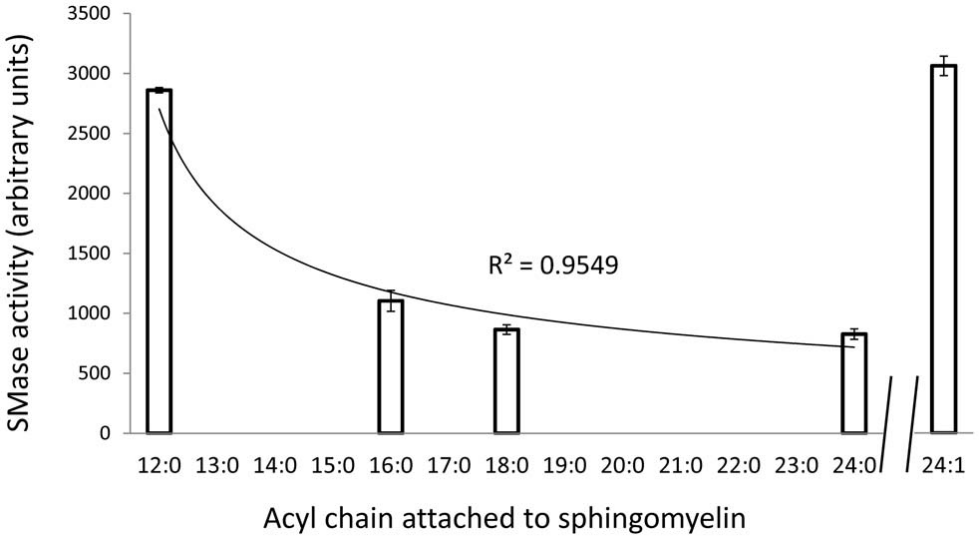
Sphingomyelinase demonstrated more specificity towards sphingomyelin with shorter fatty acyl chain. Bacterial sphingomyelinase was incubated with d18:1/12:0, d18:1/16:0, d18:1/18:0, d18:1/24:0 and d18:1/24:1 sphingomyelin and fluorescence was measured as an indicator of the enzyme specificity towards each substrate. One-way ANOVA demonstrated significant difference between specificity towards different saturated fatty acid-containing SM. p<0.001, n = 3 for each substrate. The power-fit trendline was plotted for Sphingomyelin with unsaturated acyl chain. The accuracy of the curve is more that 95% for equation y = 2704.3×^−0.517^.

To determine if sphingomyelinase enzyme could be more specific to certain SM species, we performed the sphingomyelinase assay in a cell-free system in which bacterial sphingomyelinase was incubated with SM substrate with different acyl chains (Fig. 6). We found that SM with saturated short chain acyl, such as SM(d18:1/12:0) are more specific substrates for sphingomyelinase, than SM with saturated very long chain such as SM(d18:1/24:0). This result suggests that the increase in short-chain Cer in diabetic EPCs could be due to ASM specificity to short-chain SM. Interestingly, monounsaturated long chain SM, SM(d18:1/24:1) was as specific as SM(d18:1/12:0) in the cell free assay (Fig. 6) in agreement with an increase in d18:1/24:1 ceramide in diabetic EPCs (Table 1).

### Human diabetic CD34^+^ cells treated with DHA in vitro

**Figure 7:**
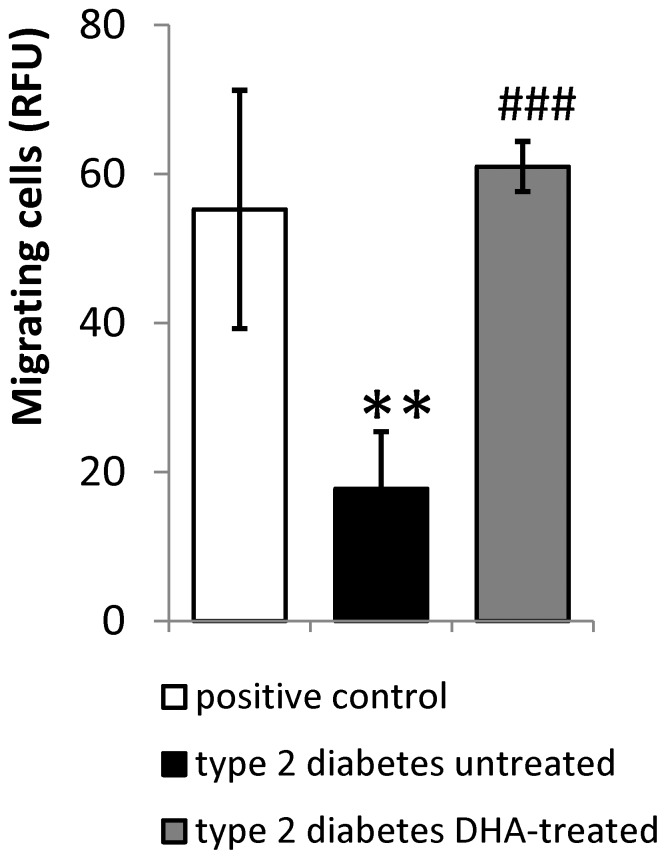
In *vitro* DHA pretreatment improves human CD34^+^ cell migration. EPCs were extracted by magnetic beads method from blood of diabetic patients. Cells were pre-treated with BSA vehicle (untreated) or 100 µM DHA and then placed in Boyden chamber for migration assay. Migration towards FBS was used as positive control. n = 4 in each group. ** = p<0.01 compared to positive control group, ^###^ = p<0.001 compared to untreated diabetic group.

To support our data from the diabetic animal model, we obtained CD34^+^ EPCs from peripheral blood of diabetic subjects. The effect of in vitro DHA treatment on EPC function was evaluated. DHA pretreatment significantly improved migration of EPCs isolated from diabetic patients compared to pretreatment with vehicle control (Fig. 7).

### Life span in diabetic animals fed DHA or control diet

**Figure 8:**
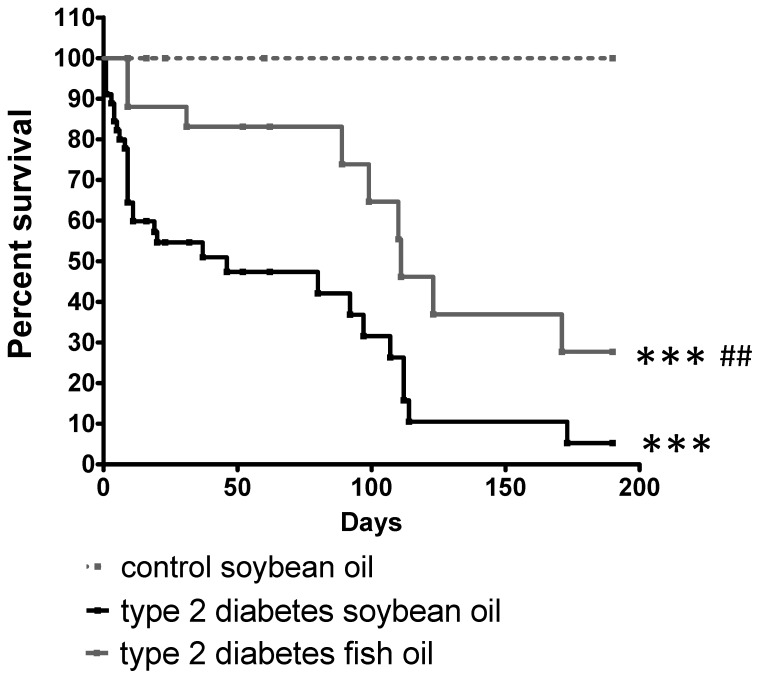
DHA-rich fish oil diet improves life span in type 2 diabetic rats. After induction of diabetes, rats were randomly assigned to soybean (black line) or DHA-rich fish oil diet (gray line) shown as day 0. Control animals (dashed line) were fed soybean oil diet. Survival was then assessed for 190 days. Diabetic rats fed DHA-rich fish oil diet had statistically improved survival as compared to soybean oil diet. n = 45 of control animals group, n = 45 of diabetic animals fed soybean oil group, n = 25 of diabetic animals fed fish oil group. *** = p<0.001 compared to control animals group and ^###^ = p<0.001 compared to diabetic animals fed with soybean oil diet.

In agreement with previous studies, diabetic animals exhibited a decreased survival rate compared to control. The DHA-rich fish oil diet, however, significantly increased the survival rate in diabetic animals (Fig. 8). The protective effect of DHA is most evident within the first 50 days of the diet. After 50 days the slopes of the survival curves were similar between diabetic animals on control or DHA-rich oil diet.

## Discussion

The metabolic insult leading to retinal vascular degeneration in diabetes is believed to initially involve endothelial cell damage due to low-grade chronic inflammation, with inadequate vascular repair by compromised bone marrow derived endothelial progenitor cells. EPCs participate in retinal vascular repair by migrating to the site of injury, by direct incorporation into vessels and primarily by providing paracrine growth factors to support to the resident vasculature. In diabetes, others and we have shown that bone marrow pathology with subsequent EPC dysfunction precedes and is necessary for development of retinal vascular degeneration [7], [8]. In this study we show that the metabolic link connecting both the initial inflammation in the retina and the dysfunctional EPCs involves activation of the central enzyme of sphingolipid metabolism, ASM.

Our previous studies in rat model of type 1 diabetes demonstrated a significant decrease in total n-3 PUFAs, especially DHA, which was tightly coupled to increased inflammatory changes in the retina [15]. A recent study by Connor *et. al.* which used the rodent model of retinopathy of prematurity demonstrated that increasing n-3 PUFAs levels by either dietary or genetic means decreased retinal TNFα levels and lessened microvessel pathology [18]. However, to our knowledge, this is the first study examining the effect of increased dietary n-3 PUFA such as DHA on development of retinopathy in type 2 diabetes model. The ultimate result of the DHA-rich dietary supplementation in this study is that it prevented retinal vessel loss as evidenced by the decreased number of acellular capillaries in retinas of type 2 diabetic animals.

It is essential to know the effect of a potential therapeutic intervention on overall physiological homeostasis in addition to focusing on the outcome for retinopathy. Besides improved retinal health, diabetic animals on the DHA-rich diet lived significantly longer than diabetic animals fed the control diet, suggesting that DHA improves more than just retinal vascular health in diabetes. Our data are in agreement with a previously published study showing the beneficial effects of dietary n-3 PUFAs on life expectancy in humans [19]. Vascular complications are the main cause of death in type 2 diabetes [20] and EPCs play a critical role in vascular regeneration [21]. We suggest that the effect of DHA on survival rate in diabetic animals could be through a generalized improvement of vascular function due to decreased vascular inflammation and enhanced vascular repair by EPCs, as observed for retinal vasculature. The results of this study suggest that the beneficial effects of DHA are due, at least in part, to inhibition of ASM activity in retinal endothelial cells and bone marrow derived EPCs.

ASM-mediated production of pro-inflammatory and pro-apoptotic Cer was shown to be an integral component of inflammatory signaling. Remarkably, we found that sphingomyelinase demonstrates more substrate specificity towards short saturated acyl chain SM as compared to very long saturated acyl chain SM, suggesting that it is more likely to produce short-chain Cer species when activated in pathological conditions such as diabetes. Indeed, we found that in diabetic EPCs, Cer and Cer metabolites containing saturated short chain acyl (14:0 and 16:0) were significantly increased. Interestingly, sphingomyelinase was also highly specific to monounsaturated very long chain 24:1-containing SM. Although its function has not been identified, levels of 24:1-containing Cer in muscles were found to be associated with insulin resistance [22]. Our results suggest that this may also be occurring in EPCs. In most cell types SM exist in concentrations that are at least an order of magnitude higher than Cer [23], thus the levels of SM do not necessarily reflect the ASM activity.

Cer enrichment of microdomains stimulates their ability to fuse [24], [25] which results in clustering of cytokine receptors and enhanced inflammatory signal transduction [9], [11]. In this study, we tested whether inflammation associated with type 2 diabetes leads to increased ASM levels in whole retina. Indeed, ASM protein level was increased more than 5-fold in diabetic retina as compared to control. Importantly, in diabetic animals fed the DHA-rich diet, levels of ASM were not elevated (Fig. 3). An increase in ASM leads to enhanced conversion of SM to Cer, followed by the activation of the key pro-inflammatory signaling pathways, including the nuclear factor kappa B (NFκB) pathway [26]. The NFkB transcription factor controls the expression of a wide range of genes, such as ICAM-1. Indeed, we found that the pattern of ICAM-1 expression follows that of ASM. Retinal ICAM-1 mRNA was increased in diabetes compared to control, but was not increased in diabetic rats fed the DHA-rich diet, highlighting the important role of ASM in diabetes-induced retinopathy. We previously observed anti-inflammatory effect of DHA through attenuating NF-κB signaling in early steps of inflammation in retinal vascular cells [27]. Based on this finding and previous observations in other models of retinopathy [18], we hypothesized that the protective effect of DHA on retinal vasculature in diabetes is mediated, at least in part, by inhibition of retinal inflammation and cytokine signaling.

In this study, mRNA levels of IL-1β, IL-6 and ICAM-1 were increased in the retinas of type 2 diabetic rats after 2 months of diabetes supporting the notion that chronic inflammation underlies the vascular pathology in type 2 diabetes. The DHA-rich diet normalized inflammatory markers to control levels and prevented endothelial cell loss and development of acellular capillaries. Although we only analyzed inflammatory markers at the transcription level in this study, others and we have previously demonstrated that there is a good agreement between mRNA and protein expression level for the markers used in this study [28], [29], [30], [31].

In addition to the reported inflammatory cytokines and adhesion molecules, we performed the measurements of a number of chemo attractants and growth factors but found no difference between control, diabetes and treatment groups. Recently others and we demonstrated that chemo attractants and growth factors controlling the release and migration of EPCs, such as SDF-1, MCP-1, VEGF are under strict circadian regulation [8], [32], [33], [34]. We believe that the variability observed in the expression level of chemo attractants was due to collection at different circadian phases which may have masked the differences between different groups.

EPCs arise from the bone marrow, migrate into the bloodstream and home to peripheral vascular beds such as skin, kidney or retina, repairing injured vasculature by incorporating directly into vessels and by providing paracrine support to the resident vasculature [21]. Diabetic patients have decreased number of circulating EPCs [35], [36] and studies by others and us suggest that EPCs isolated from diabetic patients have decreased ability to participate in endothelial tube-like structure formation compared to EPC isolated from healthy individuals. EPCs from diabetic patients are not effective in vascular regeneration due to their impaired migration and proliferation abilities [7], [8], [37], [38], [39], [40].

Our findings confirmed that in a rat model of type 2 diabetes, total numbers of EPCs in the blood were decreased. Moreover, diabetic bone marrow derived EPCs did not form colonies as well as control EPCs. Importantly, in the animals exposed to the DHA-rich diet, the number of EPCs in the peripheral blood was significantly elevated and colony formation abilities were restored (Fig. 4). This suggests that DHA stimulated EPCs proliferation and/or migratory capacity from the bone marrow into the circulation, thus increasing EPC potential to repair vasculature.

For oriented migration toward a chemotactic signal, a spatial redistribution of membrane components occurs [41]. Recent studies have highlighted the importance of compartmentalized lipids within the membrane for generating this rearrangement [41], [42]. In particular, sphingolipid-enriched microdomains play a role in membrane compartmentalization [43] and cell migration [44]. ASM has the ability to change membrane properties by converting SM to Cer. Since we previously found cellular ASM levels to be increased using *in vitro* model of diabetes [11], we hypothesized that ASM activation could also take place in EPCs in diabetes, altering sphingolipid content of EPC plasma membranes and resulting in decreased EPC migration ability. We described the inhibitory function of DHA on ASM activity in human retinal endothelial cell cultures [11] and thus proposed that this protective effect of DHA may occur on endothelial lineage cells such as EPCs. We showed that in diabetic rats, the elevation in ASM activity in EPCs was prevented by a DHA-rich diet (Fig. 5), supporting our hypothesis.

Current treatments for diabetic retinopathy are highly invasive and do not completely prevent the pathology. This study has demonstrated that dietary supplementation with the n-3 fatty acid DHA represents a safe, non-invasive, and effective strategy for prevention of diabetic retinopathy. We demonstrate for the first time that in a rat model of type 2 diabetes, DHA inhibits ASM to reduce inflammatory mediators and improve EPC function. The combined actions of DHA lead to a pronounced protective effect on retinopathy and increased life expectancy in type 2 diabetes.

## Methods

### Rat model of type II diabetic retinopathy

Bio-Breeding Zucker diabetic rat (BBZDR/Wor) rats and age-matched non-diabetic BBDR littermates were purchased from Biomedical Research Models Inc. The BBZDR/Wor strain was specifically developed as a model of type 2 diabetes. They spontaneously develop diabetes and diabetic complications including retinopathy, neuropathy, nephropathy, and macrovascular complications typical for type 2 diabetes in humans. BBZDR/Wor animals also demonstrate an early vasodegenerative stage of DR with acellular capillary development [45], thus making this model of type 2 diabetes highly applicable to human disease. All procedures involving the animal models adhered to the ARVO statement for the Use of animals in Ophthalmic and Vision research. The protocols for the animal studies were approved by the Institutional Animal Care and Use Committee at Michigan State University.

### Experimental diet

Within 1 month of diabetes onset, diabetic animals were randomly assigned to soybean of fish oil diet. AIN-93M purified rodent diet composition with 10% caloric intake as soybean oil (a standard rodent diet ingredient that contains 50.8% linoleic acid) from Dyets Inc. (Bethlehem, PA) was used as a control diet and half of the soybean oil, or 5% caloric intake was replaced with Menhaden oil (oil from a small plankton eating fish containing 10.26% DHA and 14.16% EPA) for the treatment group.

### Sphingomyelinase Assay

EPCs were lysed in acid lysis buffer (50 mM Sodium Acetate, pH = 5; 1% TritonX-100; 1 mM EDTA) with freshly added protease inhibitor cocktail (Sigma, St. Louis, MO). Sphingomyelinase activity was measured using the Amplex Red Sphingomyelinase Assay Kit (Molecular Probes, Eugene, OR) as described in the manufacturer's protocol.

Substrate specificity of sphingomyelinase was assayed in a cell-free system using the Amplex Red Sphingomyelinase Assay Kit. 4 mU of bacterial sphingomyelinase provided in the kit was incubated with 0.3 nanomoles of d18:1/12:0, d18:1/16:0, d18:1/18:0, d18:1/24:0 and d18:1/24:1 SM and the fluorescence was measured.

### Quantitative real time-polymerase chain reaction (qRT-PCR)

Total RNA was extracted from rat retinas and qRT-PCR was performed as previously described [46]. Specific primers for each gene were designed using IDT DNA PrimerQuest software (Coralville, IA). Rat gene-specific primers used in this study: IL-1β (CAAGGAGAGACAAGCAACGA and GTTTGGGATCCACACTCTCC), IL-6 (CCAGGAAATTTGCCTATTGA and GCTCTGAATGACTCTGGCTTT), TNFα (GGTCCCAACAAGGAGGAGA and GCTTGGTGGTTTGCTACGA), ICAM-1 (CCACCATCACTGTGTATTCGTT and ACGGAGCAGCACTACTGAGA). Cyclophilin was used as a control and all results were normalized to the abundance of cyclophilin mRNA.

### Human CD 34^+^ cell isolation

The study protocol was approved by the institutional review board at the University of Florida IRB #2010-411, and written informed consent was obtained from each patient. One progenitor population, CD34^+^ cells, represent a cell population with marked clinical utility [47], [48]. Circulating CD34+ cell numbers predict cardiovascular dysfunction and risk better than CD34^+^VEGFR2^+^ and CD133^+^-based populations [49], [50]. Recently, Fadini et al. [51] reported that circulating CD34^+^cell number was an independent risk biomarker of cardiovascular events and significantly correlated with outcomes in metabolic syndrome. These studies, taken together, suggest that CD34^+^ alone is sufficient as a marker for EPCs. Circulating CD 34^+^ cells were isolated from peripheral blood of 5 control and 5 diabetic patients as previously described [52]. Briefly, for each patient 150 ml of blood were collected into Sodium Citrate-containing CPT™ glass vacuum tubes (BD, Franklin Lakes, NJ) and then reacted with magnetic bead-conjugated anti-CD34 antibodies and separated according to manufacturer's directions (Stem Cell Technologies, Vancouver, BC, Canada). Clinical characteristics of the patients are presented in Table 2. After isolation, the cell were analyzed by flow cytometry for CD34 and live/dead markers using EBioscience Alexa Fluor 647 conjugated anti-human CD34 clone 4H11 antibody and EBioscience Propidium Iodine solution as quality control.

**Table 2:** Clinical characteristics of control and diabetic patients involved in the study.

Patient #	Gender	Age	Diabetes duration	HgA1C	Medications	CVD	Retinopathy	Nephropathy	Neuropathy
Patient 1	Female	46	T2D 10 years	6.8	Metformin, ACI and diet	No	Mild NPDR	Micro albuminurea, normal GFR	Yes
Patient 2	Male	59	T2D 12 years	7.0	Metformin and diet	No	Mild NPDR	Micro albuminurea, normal GFR	Yes
Patient 3	Male	60	T2D 15 years	6.7	Metformin, ACI and diet	No	Moderate NPDR	Micro albuminurea, normal GFR	Yes
Patient 4	Female	53	T2D 10 years	7.3	Metformin, actos, glyburide, statins	No	Mild NPDR	No	No
Patient 5	Male	67	T2D 5 years	6.8		No	No	No	No
Control 1	Female	47	No			No	No	No	No
Control 2	Male	56	No			No	No	No	No
Control 3	Male	58	No			No	No	No	No
Control 4	Female	42	No		statins	No	No	No	No
Control 5	Male	60	No			No	No	No	No

### Lipid Analysis by Nanoelectrospray Ionization/Tandem Mass Spectrometry

SM and Cer levels were determined by tandem mass spectrometry precursor ion mode scanning for the characteristic Cer product ion at m/z 264.4 and SM species by positive ion mode PI m/z 184 after alkaline hydrolysis of glycerophospholipds [11], [53].

### Isolation of EPC from Rat

The Rat EPCs were isolated as previously described [8]. Mononuclear cells were separated with Ficoll-Plaque Plus (GE Healthcare) gradient and resuspended in PBS containing 2% FBS and 1 mM EDTA. A custom rat negative selection kit (Stemcell Technologies Inc.) was used to deplete CD4, CD5, CD8a and OX-43 cells. Thy-1 positive cells were further extracted using the positive selection kit (Stemcell Technologies Inc.) tagged with CD90/thy-1 antibody (Abcam). Endothelial nature of isolated Thy-1 positive cells was confirmed by co-expression of CD-133, endothelial nitric oxide synthase, incorporation of Dil-acLDL and participation in capillary tube formation with human retinal endothelial cells. Countess automatic counter (Invitrogen) was used for evaluation of EPC numbers.

### Cell Migration

To test the effect of n-3 PUFA on EPC cell migration, 20,000 human CD34^+^ cells per condition were incubated in 2% fetal bovine serum-supplemented medium in the presence of either 20 mM bovine serum albumin (vehicle control), 100 µM DHA, 100 µM palmitic acid (16:0) in 5% CO_2_ at 37°C for 18 hours. After incubation, the cells were maintained overnight in StemSpan® H3000 with StemSpan® CC100 added (Stem Cell Technologies) overnight to allow the cells to recover from the isolation process. The following morning, cells were stained with Calcein-AM (Molecular Probes, Carlsbad, CA), loaded onto the upper compartment of a Boyden migration chamber (Neuro Probe, Inc. Gaithersburg, MD) and induced to migrate through a 5 µm pore-size membrane towards either 100 nM stromal cell-derived factor-1 (SDF-1), PBS (negative control), or 10% FBS (positive control). After 4 h, the number of cells that migrated was determined by measuring fluorescence emitted at 550 nm when cells were exposed to light at an excitation frequency of 485±20 nm. Each sample was run in triplicate and results expressed as percentage of migrating cells relative to positive control ± SEM.

### DHA treatment

DHA stock was prepared by dissolving fatty acid (NuCheck Prep, Inc., Elysian, MN) in ethanol to a final concentration of 100 mM. DHA was added to EPC in serum-free medium to the concentration of 100 µM fatty acid, with 20 µM charcoal-treated, solvent extracted, fatty-acid-free bovine serum albumin (BSA; Serologica Inc., Norcross, GA) which served as the fatty acid carrier. The fatty acid/albumin molar ratio was maintained at 5∶1. The final concentration of ethanol in the media was less than 0.1%. Cells were incubated with DHA for 18 hours at 37°C prior to SDF-1 treatment. Equivalent amounts of BSA (carrier control) and ethanol were added to the control plates. 16:0 (palmitic acid), and BSA was used as an additional control.

### Western blot

Proteins were extracted from rat retinas using a simultaneous extraction of proteins and RNA method as previously described [46]. Protein concentration was measured by Qubit fluorometer (Invitrogen, Eugene, OR) as described in the manufacturer protocol. Proteins were resolved on NuPAGE Novex 10% Bis-Tris gels, transferred to nitrocellulose membrane and immunobloted using anti-ASM antibody followed by secondary IRDye infrared secondary antibodies (Invitrogen, Molecular probes, Eugene, OR). Immunoreactive bands were visualized and quantified by Odyssey infrared imaging system (LI-COR Biosciences, Lincoln, NE).

### Histological assessment of retinal capillaries

Rat retinal vascular beds were isolated by trypsin digestion [54] and acellular capillaries were systemically counted in the mid-retina by two independent investigators as previously described [54].

### Statistical analysis

Data are expressed as the mean ± SD for all parameters unless specified. Factorial ANOVA with post hoc Tukey test (GraphPad Prism5, GraphPad Software, San Diego, CA) was used for comparing the data obtained from independent samples. Survival curves were compared by proc life test with Sidac multiple comparisons (SAS software, SAS Institute, Carry, NC). Significance was established at P<0.05.
